# Rational Use of Microbiological Tests in the Diagnosis of Central Nervous System Infections Using Restrictive Criteria: a Retrospective Study

**DOI:** 10.1128/spectrum.03179-22

**Published:** 2023-03-27

**Authors:** M. T. Ngo Nsoga, F. J. Pérez-Rodriguez, A. Mamin, A. G. L’Huillier, A. Cherkaoui, L. Kaiser, M. Schibler

**Affiliations:** a Division of Infectious Diseases, Department of Medicine, Geneva University Hospitals, Geneva, Switzerland; b Faculty of Medicine, University of Geneva, Geneva, Switzerland; c Laboratory of Virology, Division of Laboratory Medicine, Department of Diagnostics, Geneva University Hospitals, Geneva, Switzerland; d Pediatric Infectious Diseases Unit, Department of Women, Children and Adolescent Medicine, Geneva University Hospitals, Geneva, Switzerland; e Laboratory of Bacteriology, Division of Laboratory Medicine, Department of Diagnostics, Geneva University Hospitals, Geneva, Switzerland; University Paris-Saclay, AP-HP Hôpital Antoine Béclère, Service de Microbiologie, Institute for Integrative Biology of the Cell (I2BC), CEA, CNRS

**Keywords:** central nervous system infections, unnecessary microbiological testing, modified Reller criteria

## Abstract

Central nervous infections, mostly represented by meningitis and encephalitis, remain a diagnostic challenge despite substantial advances in microbiological tools in recent years. Meanwhile, extensive microbiological workups, which often prove to be irrelevant retrospectively, continue to be processed on a large scale, therefore leading to unnecessary costs. The main goal of this study was to evaluate a systematic approach enabling more rational use of microbiological tools in the setting of community-acquired central nervous system infection diagnosis. In this single-center descriptive study, the modified Reller criteria were retrospectively extended to all neuropathogens tested in cerebrospinal fluid (CSF) samples with the FilmArray meningitis/encephalitis panel (BioFire Diagnostics, LLC) and bacterial culture. The inclusion period was 30 months. In total, 1,714 fluid (CSF) samples analyzed from 1,665 patients over 2 and a half years were reported. According to the retrospective application of the modified Reller criteria, microbiological testing was considered unnecessary in 544 CSF samples. Fifteen positive microbiological results were found among these samples, interpreted either as inherited chromosomally integrated human herpesvirus 6 (HHV-6), a false-positive result, or a true microbial detection without clinical relevance. No CNS infection case would have been missed if these analyses were not carried out, while about one-third of all meningitis/encephalitis multiplex PCR panels would have been saved. Our retrospective analysis suggests that the modified Reller criteria could be safely applied to all microbiological tests performed in CSF, thereby saving substantial costs.

**IMPORTANCE** Microbiological testing in general and in the setting of central nervous system (CNS) infection in particular are often excessive, leading to superfluous laboratory work and costs. In this regard, restrictive criteria, named Reller criteria, have been developed to reduce unnecessary CSF herpes simplex virus 1 (HSV-1) PCR testing when suspecting encephalitis. These criteria were then adapted for increased safety to become the modified Reller criteria. This retrospective study aims at evaluating the safety of these criteria when applied to CSF microbiological testing in general, including multiplex PCR, direct examination, and bacterial culture. The postulate was that a CNS infection can be excluded if none of these criteria is present. According to our data set, no CNS infection would have been missed if the modified Reller criteria would have been applied to save microbiological tests. This study therefore proposes a simple way to reduce unnecessary microbiological testing in the context of CNS infection suspicion.

## INTRODUCTION

Central nervous system (CNS) infections cause significant mortality but also feared sequela worldwide (1, 2). Among these infections, the most frequent clinical entities are meningitis and encephalitis, which result from inflammation within the meninges and brain parenchyma, respectively. Encephalitis is frequently accompanied by some degree of meningitis, hence the term meningoencephalitis. Myelitis and radiculomyelitis are additional and less frequent forms of CNS inflammation. Infections represent the main etiology of CNS inflammation, although some meningitis cases are caused by medications, a paraneoplastic process, or an autoimmune disease. Regarding encephalitis, a significant proportion is of autoimmune origin. Among infectious causes, viruses are leading and are followed by bacteria. Fungal and parasitic causes are much rarer (3). The prevalence of these infections varies according to epidemiological factors, including age, immune status, geographic area, and other exposure types.

The diagnosis of these manifestations is based on a combination of clinical, biological, radiological, and sometimes electroencephalographic features, and microbiological tools are key to identifying an infectious etiology. These comprise direct detection tests, such as microscopy examination, culture, antigen detection, and nucleic acid detection assays, applied mostly in cerebrospinal fluid (CSF) (4).

Multiplex PCR-based commercial panels have recently been developed, allowing for the simultaneous screening of multiple neuropathogens in a short time frame, hence enabling prompt antimicrobial treatment adaptation or interruption (5–10). For instance, the FilmArray meningitis/encephalitis panel (FilmArray ME panel) (BioFire Diagnostics, LLC) can simultaneously detect 14 different targets in 1 h. On the other hand, such multiplex approaches of multiple pathogens are not based on a rational approach and are in contradiction with a step-by-step diagnostic stewardship approach; multiple simultaneous tests are often unnecessary and increase laboratory-based costs (11, 12). Indeed, these multiplex assays, and microbiological investigations in general, are often ordered as soon as a CNS infection is suspected, typically at the emergency department. However, in a substantial proportion of cases, the neurological manifestations leading to medical evaluation in an emergency room are due to a pathological process other than a CNS infection, such as migraine, confused state, fever, stroke, or various intoxications. In addition, this multiplex PCR panel includes pathogens that normally are only a concern for specific patient populations. For instance, Escherichia coli and Streptococcus agalactiae are relevant for neonates, while cytomegalovirus (CMV) and human herpesvirus 6 (HHV-6) cause encephalitis in highly immunosuppressed patients. Neither of these is a relevant pathogen to search for in immunocompetent adults. Furthermore, the sensitivity and specificity of some targets remain incompletely characterized due to the lack of extensive validation (Listeria monocytogenes, Haemophilus influenzae, Escherichia coli) (13).

One study showed a 42.7% reduction in FilmArray ME panel testing when criteria like white blood cell (WBC) count were applied, as well as a high negative predictive value of this panel (98% for all included neuropathogens and 100% for nonviral targets when the CSF WBC count was normal) (14). Another study found a 46% PCR testing reduction for enterovirus, herpes simplex 1 and 2 (HSV-1 and -2), and varicella-zoster virus (VZV) when applying acceptance criteria of 10 nucleated cells/mm^3^ in the CSF of immunocompetent adults (15).

In an attempt to reduce unnecessary testing in the setting of encephalitis suspicion, Hanson et al. have developed the so-called Reller criteria, designed to identify the cases for which the HSV-1 PCR in CSF can be safely avoided. These criteria are CSF WBC count >5/mm^3^, CSF protein level of >50 mg/dL, age of <2 years, and a history of immunosuppression (16). For patients displaying none of these criteria, the HSV-1 PCR in CSF would not be performed. Some studies confirmed the cost-effectiveness of these criteria (17, 18). However, other investigators suggested modifying these criteria in order to further decrease the risk of missed cases of HSV-1 encephalitis (19, 20). For these modified Reller criteria, the limit of WBC count has been lowered to 4 cells/mm^3^, and the age increased to 18 years, allowing for increased safety when applying these restrictive criteria (19). Our hypothesis is that these criteria could theoretically be applied to all neuropathogens searched for in CSF. Indeed, while isolated encephalitis with normal CSF values can be observed in highly immunosuppressed patients, meningitis and encephalitis in immunocompetent adults are virtually always associated with elevated CSF proteins and/or leucocytes. Furthermore, in adult immunocompetent patients, a positive microbiological result in the CSF sample with normal white blood cell count and protein content should always raise suspicion for either a false-positive or a clinically irrelevant result. Particular examples feature clinically insignificant detection of latent Epstein-Barr virus (EBV) episomes in memory B cells present in the CSF or inherited chromosomal integrated HHV-6 genomes (iciHHV-6) (21, 22).

In this retrospective observational study, we simulated the application of the modified Reller criteria to all neuropathogenic PCRs included in the FilmArray meningitis/encephalitis panel to evaluate the security and cost savings linked to this approach in a tertiary hospital. We also interpreted each positive PCR result in CSF and assessed the distribution of detected neuropathogens in various forms of CNS infections during the study period. In addition, we compared PCR panel results with reference microbiological methods.

## RESULTS

**FIG 1:**
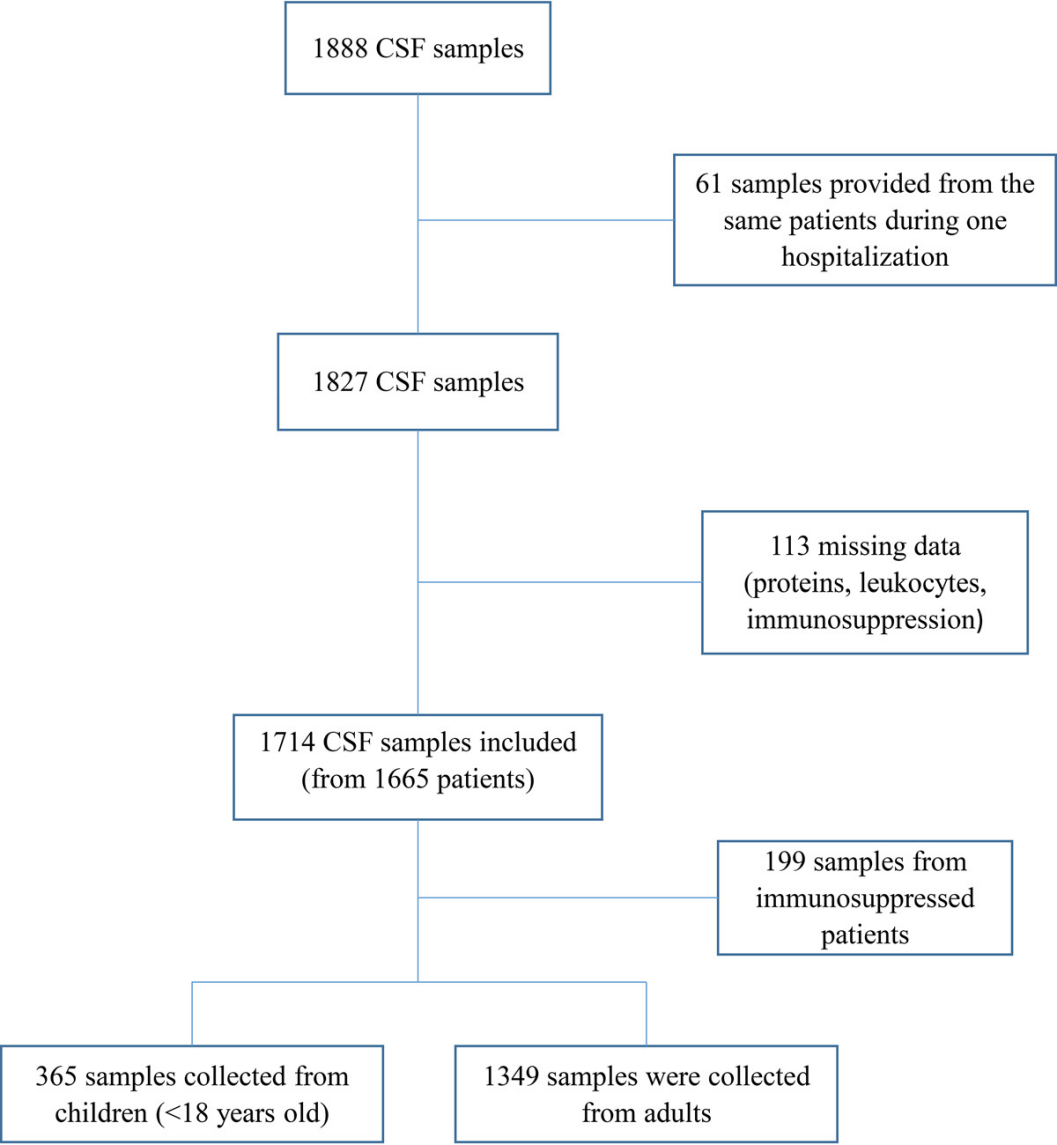
Flowchart of the study.

In total, 1,888 CSF samples were identified. We excluded 113 CSF samples with missing data precluding the assessment of the modified Reller criteria. For patients who had more than one lumbar puncture during the same hospitalization, only the first was considered, which resulted in the exclusion of 61 CSF samples. In fact, there were no situations where the first result was negative and the second was positive for the same patient. In total, 1,714 samples from 1,665 patients were included in the analysis (Fig. 1). Clinical characteristics of patients are shown in Table 1.

**TABLE 1:** Characteristics of the study population (*n* = 1,665)

Patient characteristic	Value
Age (mean [range] [yrs])	41.91 (0.003–99)
Sex	
No. female	863
No. male	802
Clinical entity (no. of patients)	
Meningitis	221
Encephalitis	83
Myelitis	14
Radiculomyelitis	3
Immunosuppression (no. of patients)	
Yes	183
No	1,482
Mean value of leukocytes (M/L)	132.27
Mean value of proteins (g/L)	0.63

Among the 227 CSF samples yielding a positive result with the FilmArray meningitis/encephalitis (ME) panel, 191 were considered to indicate a true CNS infection. Thirty-six cases with positive microbiological results in the CSF were not retained like a CNS infection. These situations were interpreted as follows: confirmed inherited chromosomally integrated HHV-6 or false-positive HHV-6 PCR result (11), HHV-6 reactivation of unclear clinical significance (7), probable inherited chromosomally integrated HHV-6 (3), false-positive Haemophilus influenzae PCR result (9), detection of VZV DNA in CSF in the context of herpes zoster ophthalmicus without meningeal irritation signs and detection of VZV DNA of undetermined significance (2), false-positive enterovirus PCR result (1), false-positive VZV PCR result (1), CSF contaminated by CMV DNA from the blood (1), and false-positive HSV-2 PCR result (1).

Several cases of codetection have been identified with the FilmArray ME panel. After reviewing the concerned patients’ medical files, a single pathogen for each case was considered to be responsible for the infection except in one case (see below). We identified 4 cases of enterovirus/HHV-6 codetection for which enterovirus was considered to be the actual neuropathogen; 1 S. agalactiae/S. pneumoniae codetection, for which the latter was regarded as the pathogen; 1 enterovirus/H. influenzae codetection, for which enterovirus was retained as the culprit; 1 enterovirus/HSV-1 codetection, for which enterovirus was considered like a pathogen; and 1 codetection of VZV and CMV, for which VZV was retained like a pathogen. There was also one enterovirus/parechovirus codetection in a newborn, for whom the two viruses were considered potential pathogens.

**FIG 2:**
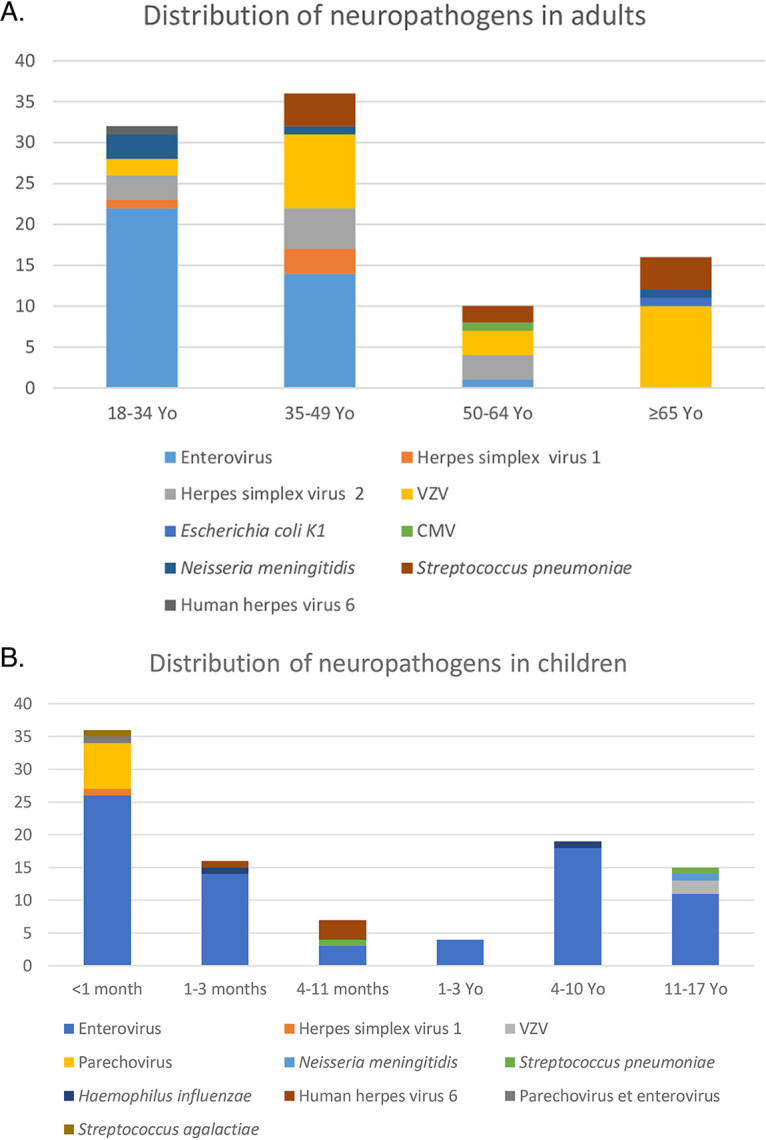
Distribution of neuropathogens in adults (A) and children (B).

Enterovirus was the most frequently identified cause of meningitis (*n* = 113). Twenty-six cases were identified in those <1 month old, 22 cases in the 18- to 34-year-old group, 18 cases in the 4- to 10-year-old group, 14 in those 1 to 3 months old, 14 in those 35 to 49 years old, 11 in those who were 11 to 17 years old, 4 in those 1 to 3 years old, 3 in those 4 to 11 months old, and 1 in the group of 50 to 64 years old (Fig. 2A and B).

The second most frequent viral neuropathogen was VZV (*n* = 26). Ten cases were found in those ≥65 years old, 9 cases in the 35- to 49-year-old group, 3 cases in the 50- to 64-year-old group, 2 cases in the 11- to 17-year-old group, and 2 in the 18- to 34-year-old group. Nine VZV CNS infections were identified in immunosuppressed patients; clinical manifestations caused by this neuropathogen were meningitis (10), encephalitis (14), and myeloradiculitis (2). HSV-2 meningitis (*n* = 11) was mostly found in young female patients, and the most affected group was the 35- to 49-year-old group (5 cases); 3 cases were found in the 18- to 34-year-old group and 3 cases in the 50- to 64-year-old group, and only 1 case of encephalitis was found. HHV-6 (*n* = 5) was most frequently detected in CSF in the group of 4- to 11-month-old children (3 cases), 1 case was found in the group of 1 to 3 months, and 1 case was found in the 18- to 34-year-old group. Clinical presentations were meningitis (3) and encephalitis (2). One of the encephalitis cases was found in a young immunosuppressed adult. Only one case of CMV encephalitis was identified during this period in an immunosuppressed elderly patient. Not surprisingly, all parechovirus cases (*n* = 7) were found in children <1 month old, and the clinical entity caused by this neuropathogen was mainly fever with possible meningitis (Fig. 2A and B).

Among bacteria detected with the FilmArray ME panel, Streptococcus pneumoniae was the most frequently found (*n* = 12); 4 cases were found in the 35- to 49-year-old age group, 4 cases in those ≥65 years old, 2 cases in the group of 50 to 64 years old, 1 case in the group of 4 to 11 months old, and 1 case in the group of 11 to 17 years old. In 10 cases, the clinical entity was meningitis, and in 2 cases, it was encephalitis. Neisseria meningitidis (*n* = 6) was the second most frequent bacteria, and 3 cases were found in young adults (18 to 34 years old), 1 case in those of the 11- to 17-year-old group, 1 case in those 35 to 49 years old, and 1 case ≥65 years old. Escherichia coli K1 was the cause of encephalitis in an elderly patient after a neurosurgical procedure. Among the 11 Haemophilus influenzae-positive monodetection results, only 2 cases of meningitis due to this pathogen were identified in children, 1 in the group of 1 to 3 months old and 1 in the group of those of 4 to 10 years old (Fig. 2A and B); 9 cases were interpreted as false positives.

Bacterial culture was performed for 1,335 CSF samples, and no positive culture result was associated with a discordant negative panel PCR result among patients with or without modified Reller criteria.

No CNS infection cases due to Listeria monocytogenes were found during the study period. One case of Mycobacterium tuberculosis meningitis was identified by culture, and one Cryptococcus neoformans meningitis case was diagnosed by antigen test in the blood and CSF. Two cases of meningitis due to Streptococcus pyogenes and Streptococcus constellatus, respectively, were also identified by culture, as well as a case of methicillin-sensitive Staphylococcus aureus (MSSA) endocarditis with CNS involvement.

**FIG 3:**
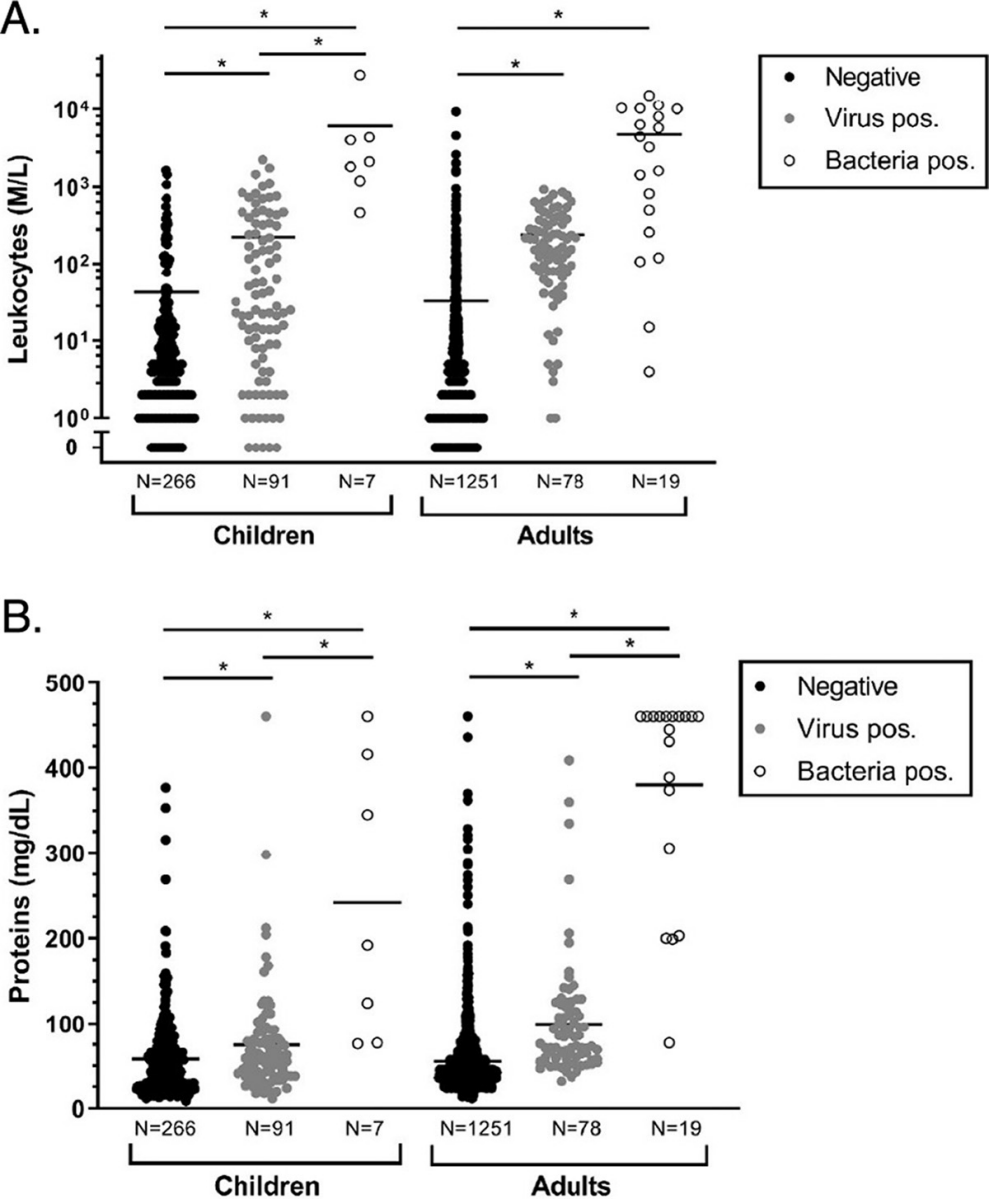
Leukocytes (A) and proteins (B) in viral and bacterial infections in both children and adults. *, *P* < 0.05.

The main biological CSF features in bacterial and viral CNS infections according to age groups are depicted in Fig. 3A and B.

Among the 1,714 CSF samples analyzed in this study, 365 were collected from children (<18 years old) and 199 from immunosuppressed patients. In total, 544 (32%) CSF samples were devoid of any of the modified Reller criteria. Among these, 15 had a positive FilmArray ME panel result, and 1 had an uninterpretable S. agalactiae result. However, none of these PCR results were considered to be associated with a CNS infection. Indeed, these results could either be explained by inherited chromosomally integrated HHV-6, nonpathogenic viral reactivation, detection of pathogen without clinical relevance, or a false-positive result (see Table S3 in the supplemental material). All of these 15 patients had an alternative final diagnosis explaining their neurological features. Thus, no acute CNS infection case was missed when using the modified Reller criteria retrospectively. Clinical retrospective data showed 10 cases with a diagnosis of pachymeningitis, encephalitis, or myelitis among these 544 samples; after analysis of the files, 2 cases were radiological pachymeningitis, 1 inflammatory and 1 either inflammatory or infectious. One cerebral abscess case already treated for 4 weeks was also identified, as well as 5 cases of autoimmune/undetermined encephalitis and 2 cases of undetermined myelitis.

Among the 544 CSF samples for which microbiological analyses were performed in the absence of modified Reller criteria, direct examination was performed in 372 of them (343 acridine and Gram staining, 24 auramine staining, and 5 Fungi-Fluor staining), and bacterial culture was done for 382 of these samples. Mycobacterial and fungal cultures were undertaken in 21 and 9 of these CSF specimens, respectively. Among positive results in this group (direct examination or culture), no case of true CNS infection was identified.

Regarding viral targets, the sensitivity of the FilmArray ME panel was 100% for each target during the study period. Low specificity was mostly observed for the HHV-6 target. VZV, HSV-1, and HSV-2 PCRs displayed a similar specificity at 99.7% (95% confidence interval [CI], 98.5 to 100) (Table S4).

Different combinations of the Reller criteria were retrospectively applied with the following findings. First, among immunocompetent adults, no CNS infection was identified for CSF samples with leukocytes <5/mm^3^, which is the upper normal limit generally used in laboratories. In contrast, among children with positive microbiological results and leukocytes <5/mm^3^, 22 cases of meningitis and or disseminated infection with possible meningitis were found. When applying only the CSF protein of ≤50 mg/dL in immunocompetent adult patients, 7 CNS infections were found. When applying both the CSF white blood cells of <4/mm^3^ and the CSF protein of ≤50 mg/dL criteria to immunosuppressed patients, one CNS infection would have been missed. When applying only the CSF white blood cells <4/mm^3^ criterion or the CSF protein ≤50 mg/dL criterion to immunosuppressed patients, we found 3 and 2 CNS infections, respectively.

The inclusion strategy of our retrospective study implies that hospitalized patients or patients not displaying a clinical presentation compatible with a CNS infection suspicion were included. Since the FilmArray ME panel is designed to detect community-acquired CNS infections, we looked at the proportion of cases among the 1,714 CSF samples of the study that actually corresponded to a disease compatible with a CNS infection that was acquired in the community and not during hospitalization. According to the information available in the medical files, 1,346 were considered community-acquired CNS infection suspicions. Among them, 414 had no modified Reller criteria (1/3); 10 had a positive FilmArray ME panel result, and 1 had an uninterpretable S. agalactiae result. As described above, none of these PCR results were considered to be associated with a CNS infection.

## DISCUSSION

The aim of this retrospective study was to evaluate the safety and effectiveness of the application of the modified Reller criteria, initially designed to reduce unnecessary HSV PCR testing (16) to microbiological testing in CSF in general in the setting of CNS infection suspicion. Initial microbiological testing in the study center included the use of the multiplex PCR FilmArray meningitis/encephalitis (ME) panel in addition to standard bacteriology and serology tests, as well as confirmatory real-time PCR assays for some viral targets (HSV-1, HSV-2, VZV, CMV, and HHV-6). Based on the analysis of 1,714 CSF samples collected from 1,665 patients from June 2017 to the end of December 2019, no CNS infection case would have been missed if these criteria would have been applied. Furthermore, approximately one-third of all ME multiplex PCR panels could have been saved, representing around 65,000 Swiss francs (CHF). In addition, approximately another 15,000 CHF could have been saved if unnecessary direct examination and bacterial and fungal cultures had been avoided. Indeed, careful analysis, including the clinical data of the 15 cases for which the CSF sample had a positive FilmArray ME panel result in the absence of any modified Reller criteria, allowed us to conclude that none of them were related to a true CNS infection. Some of these results were considered to be false-positive results, as previously described with this commercial panel (23–26).

The data yielded by this study also further characterized the reliability of the FilmArray ME panel. For bacterial targets, we did not find any false-negative results when comparing the panel results to the bacterial culture results. The microorganisms identified by culture and not detected by the PCR panel were pathogens that are not included in it. False-positive results yielded by the FilmArray ME panel essentially concerned H. influenzae, as described in other studies (24, 27). Regarding viral targets, low specificity was reported with HHV-6.

Of note, according to our data set, the CSF leukocyte count threshold could have risen to 5 cells/mm^3^ without missing CNS infection cases in immunocompetent adults, which is considered the upper normal limit in most laboratories. In contrast, when the CSF protein is ≤50 mg/dL in immunocompetent adults, some cases of CNS infections were missed.

The same general conclusion regarding the safety and usefulness of the modified Reller criteria likely applies to more restricted molecular CSF panels, as well as to standard bacteriology procedures such as microscopy examination and culture. However, this study lacks data regarding the use of the modified Reller criteria to avoid unnecessary serologic screening, especially concerning frequently used tests such as HIV, arbovirus, and spirochetes (Borrelia burgdorferi and Treponema pallidum) serologic assays.

One could argue that another limitation of this study is its retrospective character and that a future validation cohort would be needed. However, in this particular case, the data analysis was straightforward, yielding clear-cut and solid data supporting the implementation of this diagnostic stewardship strategy. The subanalysis restricted to cases of community-acquired CNS infection suspicion did not alter our conclusions. Nevertheless, the findings of this study could be confirmed by similar further studies, including prospective ones, in order to further strengthen the safety of this approach. This is particularly true for neuropathogens that were not detected in this study, such as Mycobacterium tuberculosis and Listeria monocytogenes, which are considered to be major causes of encephalitis (28).

Taken together, the data included in this study provide a basis for using restrictive criteria aiming at improving diagnostic stewardship regarding microbiological testing in the setting of CNS suspicion. Additional studies, preferably prospective ones, that include evaluation of different criteria combinations would further strengthen the usefulness and safety of this approach.

## MATERIALS AND METHODS

### Study design.

CSF samples analyzed using the multiplex PCR BioFire FilmArray meningitis/encephalitis panel (bioMérieux, Marcy l’Étoile, France) in the laboratory of virology over a 2.5-year period (from 18 June 2017 to 31 December 2019) were included in the analysis. Demographic data, CSF protein levels, and CSF cell counts were automatically extracted. Clinical data, such as admission and discharge diagnoses, other laboratory and radiologic results, and immune status, were collected manually from medical records. All files were reviewed by a medical doctor training for specialization in infectious diseases supervised by an infectious disease specialist.

The FilmArray ME panel was used according to the manufacturer’s instructions. The closed system performs nucleic acid extraction, reverse transcription, and multiplex nested PCR, automatically interprets endpoint melting curve data, and provides the result. This panel tests for seven viruses (enterovirus, HSV-1, HSV-2, CMV, VZV, HHV-6, and parechovirus), six bacteria (Escherichia coli K1, Neisseria meningitidis, Streptococcus pneumoniae, Streptococcus agalactiae, Haemophilus influenzae, and Listeria monocytogenes), and the two yeasts, Cryptococcus neoformans and Cryptococcus gattii.

Whenever available, confirmatory PCR results and bacterial and fungal culture results were compared to FilmArray ME panel results.

### Case definitions.

CNS infection referred to one of the following clinical entities: meningitis, encephalitis, encephalomyelitis, meningomyelitis, encephalomyelitis, myelitis, and radiculomyelitis.

The encephalitis definition was based on Venkatesan et al. (29); the myelitis definition was adapted from Chen et al. (30). Encephalitis and meningoencephalitis were considered a sole entity under the term encephalitis. Radiculomyelitis was defined as a combination of myelitis features and evidence of nerve root inflammation. The full description of case definitions is explicated in Table S1 in the supplemental material.

### Modified Reller criteria.

We applied the following criteria as described in reference 19. Elevated CSF white blood cell count was any white blood cell count ≥4/mm^3^. Of note, the 5/mm^3^ threshold was used for the biological meningitis definition (Table S1). Elevated CSF proteinorachia was any protein concentration >50 mg/dL. Immunosuppression refers to HIV infection with a CD4 count of <350/mm^3^, solid organ transplant with immunosuppressive treatment, hematopoietic stem cell transplant, hematologic diseases, or presence of immunosuppressive therapies, including corticosteroid therapy equivalent to prednisone >20 mg/day for more than 4 weeks.

In this study, children are defined as individuals below 18 years of age.

Cases of possible meningitis in children, i.e., like neonatal sepsis with positive viral PCR results in CSF in newborns and older children with clinical signs of meningitis but near-normal CSF analysis, were considered meningitis.

Every positive microbiological result was interpreted by an infectious disease physician by reviewing medical files in order to distinguish true CNS infection from clinically insignificant results. In cases of doubt, the files were reviewed by an additional infectious diseases physician in order to decide.

Bacteriological testing details are available on request; technical details regarding in-house PCR assays are included in Table S2.

### Statistical analysis.

All analyses were performed using R software version 4.1.0 and GraphPad Prism 9.

The Mann-Whitney test was used to calculate *P* values. Statistical significance was defined as a *P* value of <0.05. Sensitivity and specificity were calculated for each viral target, and their 95% CIs were calculated according to the Wilson-Brown method using GraphPad Prism version 9.3.1.

### Ethical considerations.

The Cantonal Ethics Commission for Research on Human Beings (CCER, Geneva, Switzerland) approved the submission for publication of this retrospective analysis performed in the setting of a quality project within the Geneva University Hospitals.
