# Supplementary material for: Rational Use of Microbiological Tests in the Diagnosis of Central Nervous System Infections Using Restrictive Criteria: a Retrospective Study
Source: Microbiol Spectr. 2023 Mar 27;11(2):e03179-22. doi: 10.1128/spectrum.03179-22 (PMC10100671; doi:10.1128/spectrum.03179-22)
Supplement: Supplemental file 1 — Supplemental material. Download spectrum.03179-22-s0001.pdf, PDF file, 0.3 MB [file spectrum.03179-22-s0001.pdf]

Supplemental table 1. Criteria defining encephalitis, meningoencephalitis, meningitis, myelitis and meningomyelitis

<table>
<tr><th>Encephalitis and Meningo-encephalitis</th><th>Meningitis</th><th>Myelitis and Meningomyelitis</th></tr>
<tr>
<td><u>Major criteria</u>(required)
Altered mental status (defined as decreased or altered of consciousness, lethargy or personality change) lasting ≥24 hours with no alternative cause identified

<u>Minor criteria</u> (2 required for possible encephalitis, ≥3 required for probable or confirmed encephalitis)
Documented fever ≥38,2°C within the 72 hours before or after presentation
Generalized or partial seizures not fully attributable to a preexisting seizure disorder
New onset of focal neurologic findings
CSF leukocyte count ≥5M/L
Abnormality of brain parenchyma on neuroimaging suggestive of encephalitis
Abnormality on EEG that is consistent with encephalitis and not attributable to another cause</td>
<td><u>≥ 2 following criteria</u>
Headache
Fever ≥ 38,2°C
Photo- and/or Phonophobia
Neck stiffness

AND

CSF leukocyte count ≥5M/L

Notes:
Diagnosis of meningo-encephalitis is considered if clinical, radiological or biological findings is suggestive of meningeal inflammation
In children, only clinical signs were considered; in newborns and small children under 3 months, the major criteria inclusion for meningitis was febrile event without clinical focus</td>
<td><u>Major criteria</u> (≥ 1 required)
Asymmetrical flaccid weakness with reduced or absent reflexes or sensory symptoms or signs
Hyper intensities of the spinal cord on T2 weighted MRI imaging
<u>Minor criteria (optional)</u>
Fever ≥ 38,2°C
CSF leukocyte count ≥5M/L</td>
</tr>
</table>

Supplemental table 2. In-house and commercial real-time PCR assays details.

Target	Primer/Probe	Sequence	Final Conc. nM
**HSV1/2**	Forward	5'- CCGTCAGCACCTTCATCGA -3'	300
	Reverse	5'-CGCTGGACCTCCGTGTAGTC -3'	300
	Probe	5'-FAM-CCACGAGATCAAGGACAGCGGCC-TAMRA-3'	100
**HSV Typisation**	Forward	5'-CGCATCAAGACCACCTCCTC-3'	900
	Reverse	5'-GCTCGCACCACGCGA-3'	600
	Probe HSV1	5'Vic-TGGCAACGCGGCCCAAC-TAMRA3'	100
	Probe HSV2	5'FAM-CGGCGATGCGCCCCAG-TAMRA3'	100
**VZV**	Forward	5'- CGG CAT GGC CCG TCT AT -3'	600
	Reverse	5'-TCG CGT GCT GCG GC -3'	600
	Probe	5'-FAM ATT CAG CAA TGG AAA CAC ACG ACG CC TAMRA-3'	200
**Enterovirus**	Forward	5'-GCTGCGYTGGCGGCC-3'	600
	Reverse	5'-GAAACACGGACACCCAAAGTAGT-3'	900
	Probe	5'-FAM-CTCCGGCCCCTGAATGYGGCTAA-TAMRA-3'	200
**Parechovirus**	Forward	5'-CTGGGGCCAAAAGCCA-3'	900
	Reverse	5'-GGTACCTTCTGGGCATCCTTC-3'	900
	Probe	5'-VIC-AAACACTAGTTGTAHGGCCC-3'-MGB -3'	200
**CMV**	Forward	5'-GATCCGCTGACGCGTTTG -3'	900
	Reverse	5'-GCCGCCAGTCGTAACGAT -3'	900
	Probe	5'-FAM-TCATCGATCGGCGGATCACCAC-TAMRA -3'	300
**HHV-6 (A+B) ql**	Forward	5'-GACAATCACATGCCTGGATAATG -3'	600
	Reverse	5'-TGTAAGCGTGTGGTAATGGACTAA -3'	600
	Probe	5'-FAM-AGCAGCTGGCGAAAAGTGCTGTGC-TAMRA -3'	200
**HHV6 qPCR**	HHV6 R-Gene (Biomérieux, ref 69-006B), conditions according to manufacturer		

PCR mix for DNA viruses: TaqMan^{TM} Universal PCR MasterMix (Thermofisher, ref 4304437)
PCR mix for RNA viruses: Quantitect Probe RT-PCR Kit (Qiagen, ref 204443)

Run conditions: 50°C - 30' (Reverse transcription for RNA viruses)
95°C - 15'
45 x (94°C - 15" / 60°C - 1')

Supplemental Table 3. Interpretation of positive multiplex panel PCR results in CSF from patients without modified Reller criteria

Sex	Age (years)	Leukocytes / mm^3	Protein (mg/dL)	Filmarray result	Immonusuppression	Initial diagnosis	Result interpretation	Final diagnosis
M	48	<3	40	HHV-6	No	Agitation on alcohol intoxication and cocaine	Chromosomal integration to HHV-6	Acute poisoning from alcohol and cocaine consumption
M	48	2	48	HHV-6	No	Suspected stroke	Probable chromosomal integration to HHV-6	Migraine-like headache
M	25	1	26	HHV-6	No	Fever after returning from a trip	Weak HHV-6 reactivation of undetermined significance in immunocompetent	Probable secondary syphilis
M	36	1	30	HHV-6	No	Severe sepsis of undetermined origin, probably urinary due to pigtail obstruction	Low HHV-6 detection of undetermined significance	Disorders of the state of consciousness
M	25	1	31	HHV-6	No	Management of an inaugural crisis	False positive	Suspicion of right temporal epilepsy, of non-lesional origin
F	28	<1	42	VZV	No	Varicella	Detection of VZV of undetermined significance	Varicella
M	43	2	47	Enterovirus	No	Left facio-brachio-crural motor hemi-syndrom	False positive	Right posterior capsulo-caudo-lenticular ischemic stroke in SAPL setting
F	48	1	37	HHV-6	No	Severe or unusual headaches	Detection of low amount of HHV-6 DNA of undetermined significance	headaches
F	45	<1	38	VZV	No	viral meningoencephalitis	False positive	Headaches
F	67	<1	28	HHV-6	No	transient ischemic attack	Probable chromosomal integration with HHV-6	Suspicion of transient ischemic stroke
F	22	<1	28	*H.influenzae*	No	Transfer for suspicion of meningitis	False positive	Febrile headaches
F	91	3	34	Not interpretable *S.agalactiae*	No	Disorders of the state of consciousness	Not interpretable *S. agalactiae*	Acute confusional state linked to urinary tract infection (*P. aeruginosa*)
F	60	3	36	HHV-6	No	Febrile state with headache, vomiting	Chromosomal integration to HHV-6	flu syndrome
F	59	1	39	HHV-6	No	Suspected stroke	HHV-6 reactivation of undetermined significance in immunocompetent	Dizziness and gait instability in the context of decompensated diabetes
F	73	2	31	*H.influenzae*	No	Disorder of the state of consciousness	False positive	Possible encephalitis of undetermined origin
F	69	2	42	HHV-6	No	ischemic stroke	Chromosomal integration to HHV-6	Ischemic stroke of the right corona radiata of probably micoangiopathic origin

Supplemental table 4. Virologic Filmarray ME panel results compared to single-target real-time PCR results, when available.

	**Sensitivity**		**Specificity**		**PPV**	**NPV**
	**TP/(TP+FN)**	**% (95% CI)**	**TN/(TN+FP)**	**% (95% CI)**	**% (95% CI)**	**% (95% CI)**
**Parechovirus**	6/6	100% (61.0–100)	42/42	100% (91.6–100)	100% (61.0–100)	100% (91.6-100)
**HHV-6**	24/24	100% (86.2–100)	8/12	66.7% (39.1–86.2)	85.7% (68.5–94.3)	100% (67.6-100)
**VZV**	28/28	100% (87.9–100)	370/371	99.7% (98.5–100)	96.5% (82.8–99.8)	100% (99-100)
**CMV**	3/3	100% (43.8–100)	9/9	100% (70.1–100)	100% (43.8–100)	100% (70.1-100)
**Enterovirus**	20/20	100% (83.9–100)	16/17	94.1% (73–99.7)	95.2% (77.3-99.8)	100% (80.6-100)
**HSV-1**	5/5	100% (56.5–100)	376/377	99.7% (98.5–100)	83.3% (43.6-99.1)	100% (99-100)
**HSV-2**	11/11	100% (74.1–100)	376/377	99.7% (98.5–100)	91.7% (64.6-99.6)	100% (99-100)

The 95% CI was calculated according to the Wilson-Brown method using GraphPad Prism Version 9.3.1.
